# Live Cell Imaging of Enzymatic Turnover of an Adenosine 5′-Tetraphosphate Analog

**DOI:** 10.3390/ijms22168616

**Published:** 2021-08-10

**Authors:** Anayat Bhat, Shuang Li, Daniel Hammler, Martin J. Winterhalder, Andreas Marx, Andreas Zumbusch

**Affiliations:** Department Chemie, Universität Konstanz, 78457 Konstanz, Germany; anayat.2.bhat@uni-konstanz.de (A.B.); shuang.2.li@uni-konstanz.de (S.L.); daniel.hammler@uni-konstanz.de (D.H.); martin.winterhalder@uni-konstanz.de (M.J.W.)

**Keywords:** ATP turnover, ATP probe, live cell microscopy, fluorescence microscopy, FRET

## Abstract

The hydrolysis of nucleotides is of paramount importance as an energy source for cellular processes. In addition, the transfer of phosphates from nucleotides onto proteins is important as a post-translational protein modification. Monitoring the enzymatic turnover of nucleotides therefore offers great potential as a tool to follow enzymatic activity. While a number of fluorescence sensors are known, so far, there are no methods available for the real-time monitoring of ATP hydrolysis inside live cells. We present the synthesis and application of a novel fluorogenic adenosine 5′-tetraphosphate (Ap4) analog suited for this task. Upon enzymatic hydrolysis, the molecule displays an increase in fluorescence intensity, which provides a readout of its turnover. We demonstrate how this can be used for monitoring cellular processes involving Ap4 hydrolysis. To this end, we visualized the enzymatic activity in live cells using confocal fluorescence microscopy of the Ap4 analog. Our results demonstrate that the Ap4 analog is hydrolyzed in lysosomes. We show that this approach is suited to visualize the lysosome distribution profiles within the live cell and discuss how it can be employed to gather information regarding autophagic flux.

## 1. Introduction

Adenosine triphosphate (ATP) is best known for being the universal energy carrier in biological cells. Its hydrolysis yields the Gibbs free energy needed to drive otherwise endergonic reactions [1]. In many other cellular processes, however, ATP is also used in different manners. For example, it serves as a substrate for the synthesis of the second messengers cAMP (cyclic adenosine monophosphate) [2] and cyclic-di-AMP [3], it is utilized as a cofactor for kinases, and it plays a pivotal role in the posttranslational phosphorylation of proteins [4]. Already, these few examples show the broad range of functions that ATP fulfills in cells. Methods to visualize the enzymatic ATP turnover in live cells therefore promise to give insight into a broad variety of cellular processes by the monitoring of ATP coupled enzymatic activity. Established techniques for measuring ATP turnover include radioactive labeling of ATP [5] or the spectroscopic detection of phosphate released during ATP hydrolysis. The latter can be achieved by the formation of molybdenum blue or the formation of complexes with malachite green [6]. Alternatively, the formation of ADP can be detected with enzymatic assays [7,8]. However, all of the aforementioned processes require the purification of the reaction products before analysis, such that they do not lend themselves to the real-time and continuous measurement of ATP hydrolysis.

A promising alternative to these approaches is the use of fluorescent ATP analogs that change their spectroscopic properties upon hydrolysis. Recently, we reported the synthesis of doubly fluorescently labelled diadenosine triphosphate (Ap3A) [9] and adenosine tetraphosphate (Ap4) [10] analogs, in which one fluorophore is attached to one nucleotide, whereas the other fluorophore is located at the other nucleotide or the δ-phosphate, respectively [11]. The fluorophores were chosen such that they undergo Förster resonance energy transfer (FRET) in the intact analog. Upon cleavage, the two fluorophores are separated from each other, which interrupts FRET and leads to significant changes in the fluorescence emission properties, that can be used as a spectroscopic read-out. It is evident that an impairment of the enzymatic acceptance of the analogs due to the bulky modifications represents a general problem in the use of fluorophore modified ATP analogs. Nevertheless, we showed that an Ap3A analog of this type is processed by the fragile histidine triad protein FHIT [9] and that a doubly labelled Ap4 analog is cleaved by the human Ap4A hydrolase NudT2 [10]. In the latter case, we used the cyanine fluorophores Cy3 and Cy5 as a FRET pair, which allowed for cellular imaging of the turnover of the Ap4 analogs after fixation of the cells.

In this contribution, we report the synthesis of a new Ap4 analog and the monitoring of its enzymatic turnover in live cells. Instead of using two fluorophores, we designed a construct in which the fluorescence of one fluorophore was quenched by FRET to a non-fluorescing chromophore. Upon the enzymatic cleavage of this compound, FRET ceased and strongly increased the fluorescence of the fluorophore to be detected. Fluorescence spectroscopy of the compounds in the presence of different inhibitors showed that the novel Ap4 analog was indeed enzymatically processed. Confocal fluorescence microscopy was used to demonstrate that this also occurred in live cells. Detailed colocalization studies with various markers revealed that the main hydrolytic activity was located in the lysosomes. These findings were corroborated by monitoring the impact of different inhibitors of the lysosomal activity. Our imaging data further show that the hydrolysis of the Ap4 analogs exhibited a quantitative correlation with cellular autophagy. We present evidence that the Ap4 analogs are suitable for visualizing lysosome distribution profiles and autophagic flux in live cells.

## 2. Results and Discussions

### 2.1. Synthesis of an Ap4 Analog as Hydrolysis Reporter

**Figure 1:**
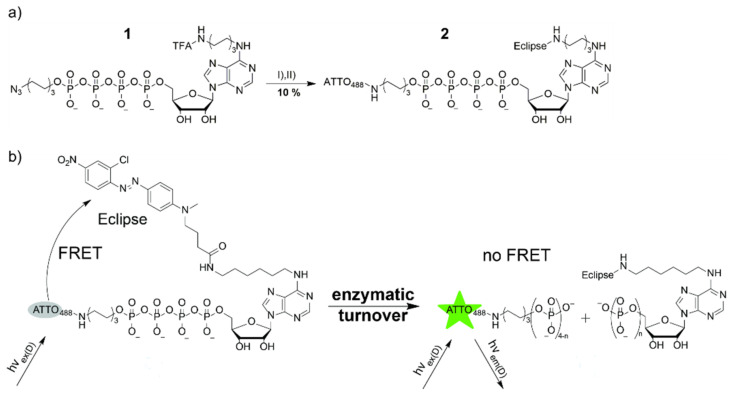
(**a**) Synthesis of the Ap4 analog **2**. Conditions: (**I**) 1. aqueous ammonia (7.5%), 5 h at RT. 2. Eclipse-NHS 1 M NaHCO_3_ (pH = 8.7), DMF/water (4:1), RT for 1 h, 29 %; (**II**) 1. TCEP-HCl, water/meoh/tri-ethylamine (7:8:4), on, at RT. 2. NaHCO_3_ (pH = 8.7), water, ATTO488-NHS, RT for 1 h, 33 %. (**b**) Principle of operation of the Ap4 analog: In the intact analog, the fluorescence of the donor ATTO488 is quenched by the acceptor Eclipse via FRET. Upon cleavage, spatial separation of the donor and acceptor leads to ATTO488 fluorescence emission.

We used the hydrophilic donor fluorophore ATTO488, which has a long fluorescence lifetime of τ = 4.1 ns and a fluorescence quantum yield of 80% [12]. This donor was attached to the δ-phosphate of adenosine tetraphosphate **1** via a phosphor ester. As an acceptor chromophore, we synthesized the non-fluorescent quencher eclipse and attached it to the N6 position of the nucleobase (Figure 1). In this way, we obtained the Ap4 analog **2**, which could be used as a turn-on probe with increased fluorescence upon cleavage. The appearance of strong fluorescence was later the basis for the monitoring of the molecular probe’s cleavage by ATP hydrolyzing enzymes.

To synthesize compound **2**, we used an orthogonally protected adenosine tetraphosphate **1** with an azide and a trifluoroacetyl (TFA) residue. This protocol was already employed earlier by us to synthesize doubly labeled ATP derivatives [11]. Treatment of compound **1** with an aqueous ammonia solution yielded the mono deprotected analogue, which was reacted with the eclipse-N-hydroxysuccinimide (NHS) ester at pH = 8.7 in a water/DMF mixture (29% yield). In the next step, azide was reduced with tris(2-carboxyethyl)phosphine hydrochloride (TCEP) in a Staudinger type reaction. ATTO488-NHS ester was synthesized from phthalic acid and 3-aminophenol [13], and coupled at pH = 8.7 in water to yield compound **2** (33% yield).

### 2.2. Enzymatic Cleavage of the Ap4 Analog

The stability of the Ap4 analog against pH changes is a prerequisite for its intended use in live cells. With our target compound **2** in hand, we therefore tested its stability at different pH values. To ensure a stable pH value during the experiments, we used pH = 2 and 10 for the glycine buffer, pH = 4 for the acetate buffer, and pH = 7 for the phosphate buffer, with a final concentration of 50 mM each, and incubated compound **2** for 2.5 h at 37 °C. Analytical RP-HPLC revealed that only for pH = 2 was a small amount of compound **2** (4%) hydrolyzed during the incubation time (cf. Supplementary Materials). For all other pH values, no degradation over time was observed. We performed similar experiments by monitoring the fluorescence intensities of 20µM of **2** in 50 mM buffer solutions in the respective buffers at pH = 2, pH = 7, and pH = 10. In all of the cases, the observed fluorescence intensity changes over 60 min were below 8%, which confirmed the high stability of compound **2** with respect to pH changes (cf. Supplementary Materials).

**Figure 2:**
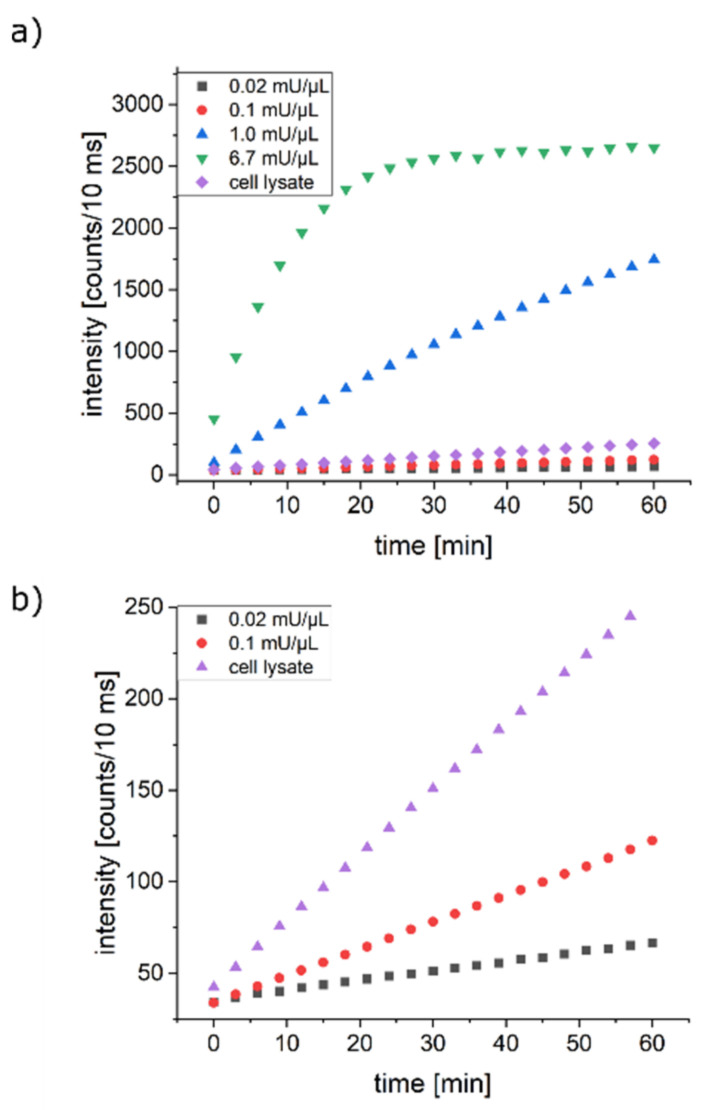
(**a**) Fluorescence intensity changes with time of the Ap4 analog 2 in a buffered solution with different concentrations of snake venom phosphodiesterase I (SVPD) and in cell lysate. (**b**) Zoom into the lower intensity region of (**a**).

To check whether **2** was suited as a model to monitor ATP hydrolysis in live cells, we investigated its cleavage under various conditions. As an initial check for the acceptance of the Ap4 analog as a substrate for enzymatic reactions, we monitored its behavior in presence of snake venom phosphodiesterase I (SVPD), an enzyme known to cleave a very broad range of modified nucleotides [11]. An analysis of the cleavage products via RP-HPLC showed complete conversion of the analog (cf. Supplementary Materials). Based on this finding, we monitored the fluorescence intensity of **2** in solutions with varying concentrations of SVPD. At high concentrations of SVPD, we observed a strong increase of fluorescence intensity, which levelled off in a plateau after roughly 10 min (Figure 2), which confirmed the HPLC results. The cleavage of **2** led to the spatial separation of the donor and quencher, which resulted in the observed increase in fluorescence emission. Lowering the concentration of SVPD in the solutions led to slower reaction kinetics. As SVPD is very tolerant towards modifications of its substrates, we tested whether the Ap4 analog was also cleaved under more restrictive conditions. To this end, we also investigated the fluorescence intensity changes of **2** in HeLa cell lysates. We found an increase of fluorescence intensity with time, the kinetics of which was faster than those of the lower concentrated SVPD solutions tested here (Figure 2). To gain more insight into the underlying photophysics, we also measured the fluorescence lifetimes changes of **2** with time in a SVPD solution. Shortly after the dissolution of **2** in the medium, the fluorescence decays were biexponential, with a fast component at around 500 ps and a slow component at 4.1 ns. Whereas the short component was attributed to the quenched emission of ATTO488 in intact **2**, the slow component corresponded to the unquenched emission of ATTO488. Even at low concentrations of SVPD, after a few minutes, the emission was dominated by the unquenched dye, such that the emission data were well fitted with a monoexponential decay with τ = 4.1 ns (data not shown).

### 2.3. In Vitro Inhibition of the Ap4 Analog Cleavage

**Figure 3:**
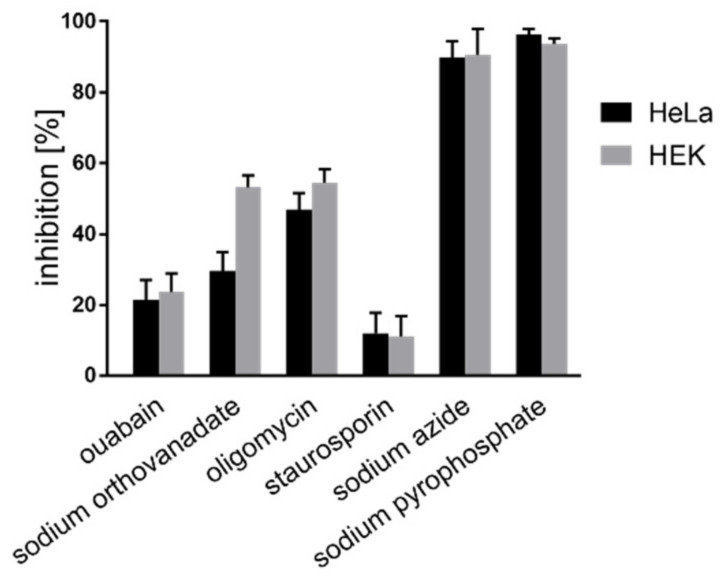
In vitro inhibition of Ap4 cleavage in HeLa and HEK cell lysates by various inhibitors monitored by fluorescence intensity measurements.

The results of the experiments just described provide strong indications that the Ap4 analog **2** is cleaved enzymatically. To prove this further and to potentially get insight into the classes of enzymes that hydrolyze **2**, we performed in vitro assays with specific inhibitors of various prominent cellular ATP consuming pathways. The inhibitors used were ouabain, an inhibitor of the sodium-potassium ATPase pump [14]; oligomycin and NaN_3_, the inhibitors of the mitochondrial F0-F1 ATPase system [15]; staurosporine, a protein kinase inhibitor [16]; sodium orthovanadate, an inhibitor of phosphatases and kinases [17]; and Na_4_P_2_O_7_ which acts as a competitive inhibitor of ATPases [18]. The hydrolysis of the Ap4 analog was monitored as the fluorescence increase in a plate reader assay at 37 °C. Cell lysates were prepared from two different cell lines, HeLa and HEK293T. A wide range of inhibitor concentrations was used and the kinetics were monitored. The increase in the concentration of inhibitors in each case resulted in the inhibition of the hydrolysis in different proportions. Very strong inhibition was seen for NaN_3_ and Na_2_P_2_O_7_, while in all of the other cases, weaker, but still pronounced, decreases in the hydrolysis of **2** were observed. (Figure 3). These findings further hint at the enzymatic cleavage of the Ap4 analog **2**.

### 2.4. Live Cell Imaging of the Ap4 Analog

As the Ap4 analog itself is not cell-permeable, we next evaluated membrane permeabilisation using the nonionic surfactant Triton-X-100 and electroporation as two experimental strategies for its internalization in live cells. In the first type of experiments, HeLa cells were treated with a mixture of 0.01% Triton X-100 and 100 μM of the Ap4 analog in 1X phosphate-buffered saline (PBS) for 12 min at 4 °C. After permeabilization, the cells were washed with 1X PBS and then imaged. It is known that low concentrations of Triton-X-100 permeabilize the plasma membrane by insertion into the lipid bilayer [19]. Prior to permeabilization, the cells were incubated for 30 min at 4 °C to slow down their metabolism. This prevented the cleavage of the Ap4 before the starting the imaging on the microscope. Using this procedure, we observed internalization of the constructs into the cells in point-like structures. However, some of the cells treated with Triton-X-100 collapsed during the experiment, and the overall viability decreased due to a gradual disintegration of the plasma membrane (data not shown). We therefore tested electroporation as a different internalization approach. Using the protocol detailed in Materials and Methods, we found that the cells treated with the Ap4 analog exhibited little morphological changes upon electroporation. The viability assays of the cells electroporated in the presence of the Ap4 analog also showed only an insignificant decrease in viability a few hours after the treatment (cf. Supplementary Information). In all of our experiments, we therefore employed electroporation as the method for the delivery of the Ap4 analog into cells.

**Figure 4:**
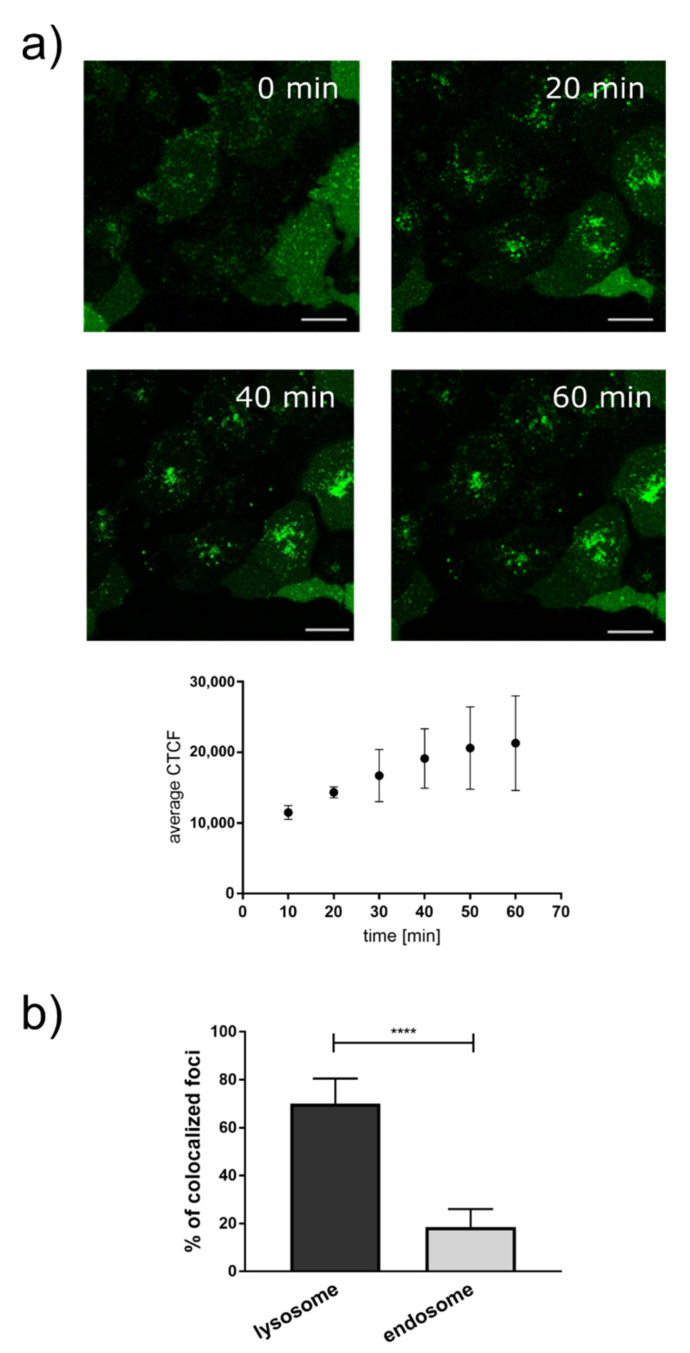
(**a**) Representative images of the increase in the Ap4 fluorescence intensity (green) in live HeLa cells as a function of time; scale bar: 20 µm. The plot below shows the quantitative average increase in the corrected total cell fluorescence (CTCF) with respect to time. The values indicate the average of 20 cells and the error bars indicate the standard deviation of the mean. (**b**) Comparison of the colocalization of Ap4 hydrolysis with lysosomes and that of endosomes. Error bars represent the SD (N = 20). Statistical significance determined by one-way ANOVA, **** *p* < 0.0001.

In order for it to be useful as a probe for cellular ATP consuming processes, the Ap4 analog needs to be cleaved in live cells after successful internalization. Cleavage would result in an increase in the fluorescence signal useful as a readout for following ATP dependent enzymatic processes. To test whether the Ap4 analog is processed in this manner, we followed the change of intensity of the Ap4 analog internalized into HeLa cells using electroporation by continuous confocal fluorescence microscopy. Already at the first images taken after electroporation, we observed Ap4 fluorescence in punctuate structures (Figure 4a). We attributed this to the time lag between the electroporation and the fluorescence imaging, such that already in the first images cleaved Ap4 was detected. The detected intensity clearly increased on a time scale of tens of minutes, proving that Ap4 is indeed processed in live cells.

### 2.5. Intracellular Localisation of the Ap4 Analog

As depicted in Figure 4a, immediately after electroporation and replacement of the electroporation buffer with the complete medium, the confocal fluorescence microscopy images showed fluorescence of the Ap4 analog appearing in punctuate structures in live cells. The emergence of fluorescence already hinted at the hydrolysis of the analogs. To rule out that these observations were due to **2** being taken up by the cellular endosomal pathway for non-specific degradation, we did a colocalization analysis of **2** with the 70 kDa Dextran-Rhodamine B, a widely used endocytosis marker [20,21,22,23]. The very poor colocalization of **2** with endosomes compared with that of the lysosomes indicates that **2** is not taken up by the cell non-specifically by endocytosis, and is not hydrolyzed through endosomal pathways (Figure 4b).

**Figure 5:**
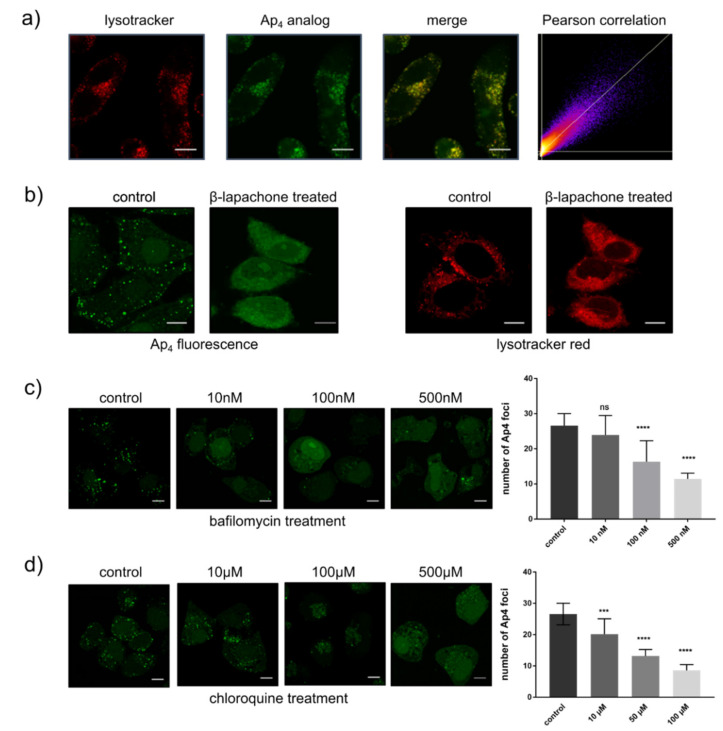
Colocalization of the Ap4 analog with lysosomes and te heffect of β-lapachone on integrity lysozomes and Ap4 analog hydrolysis. (**a**) HeLa cells were treated with lysotracker (**red**) and Ap4 analog (**green**). Colocalization of both compounds is shown in yellow. A scatterplot of red and green pixel intensities is shown. The average Pearson’s correlation coefficient for 10 cells is 0.91 (SD = 0.015). Scale bar: 10 µm. (**b**) Fluorescence images showing the intensity distribution of the Ap4 analog and lysotracker before and after the treatment of cells with β-lapachone. Effect of bafilomycin (**c**) and chloroquine (**d**) on Ap4 hydrolysis. Statistical significance compared with the control. Ordinary one-way ANOVA *** *p* < 0.001, **** *p* < 0.0001; ns: not significant. Scale Bar: 10 μm.

To further unravel the nature of the cellular structures in which the Ap4 analog was located, we performed confocal fluorescence microscopy colocalization experiments using the organelle stains mitotracker and lysotracker. Colocalization with a strong correlation (Pearson’s correlation factor 0.91) was found between the Ap4 analog hydrolysis puncta and lysosomes (Figure 5a). This implies that the hydrolysis of the Ap4 analog takes place in the lysosomes. To further test this hypothesis, we performed experiments on cells treated with β-lapachone. β-lapachone damages lysosomes and induces necrosis by elevating the levels of free radicals [24,25]. Upon β-lapachone treatment of cells previously electroporated with the Ap4 analog, we observed a significant decrease in punctuate fluorescent structures. At the same time, fluorescence increased in the cytoplasm (Figure. 5b). This is a second indication that lysosomes are prominently involved in the hydrolysis of the Ap4 analog.

To further test whether the hydrolysis of the Ap4 analog occurs in lysosomes, two lysosomal inhibitors were used, as follows: Chloroquine is a lysosomotropic agent that inhibits the enzymatic activity by increasing the pH inside the lysosomes. Its monoprotonated form diffuses into the lysosomes, where it is deprotonated and trapped, which changes the lysosomal pH, thereby inhibiting the lysosomal activity [26]. By contrast, bafilomycin A1 is a macrolide antibiotic that is used as an inhibitor of lysosomal H^+^-ATPase [27,28]. Bafilomycin prevents the acidification of endosomes and lysosomes, and thus inhibits lysosomal functioning, including autophagic flux [29,30]. In the experiments, the cells were treated with increasing concentrations of the respective inhibitors overnight, and the Ap4 analog was added and internalized via electroporation before the cells were imaged. The use of either inhibitor led to a drastic decrease in the hydrolysis of the Ap4 analog in live cells (Figure 5c,d). This is an additional indication that the Ap4 analog is indeed hydrolyzed in lysosomes.

The fact that the Ap4 analog **2** is located and cleaved in the lysosomes raises the question of whether **2** can be used to follow the cellular ATP consuming processes involving lysosomes. As one exemplary cellular process, we investigated whether the cleavage of the Ap4 analog **2** can be related to the autophagy. This choice was made as lysosomes play a fundamental role in the cellular autophagy, and because previous reports showed that all the different variants of autophagy require significant amounts of ATP [31]. To this end, we transiently expressed the fusion protein LC3B-red fluorescent protein (RFP) in HeLa cells and cultured the cells for 24 h. The LC3B protein normally resides in the cytosol. During autophagy, it is conjugated to phosphatidylethanolamine and then associates with phagophores. This makes it useful as an autophagy marker [32]. In the experiments, the cells were additionally treated with Ink-128 (sapanisertib), an autophagy inducer, or chloroquine, an autophagy inhibitor, or with nothing as the control. Sapanisertib is a potent and selective inhibitor of mTOR that induces autophagy through the dysregulation of certain metabolic pathways [33]. Cells were imaged after electroporation with the Ap4 analog.

**Figure 6:**
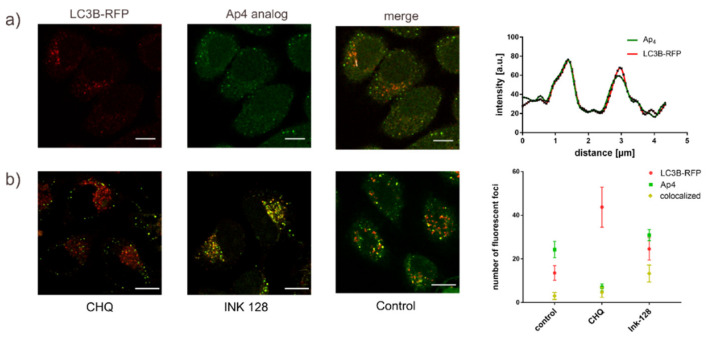
Colocalization of Ap4 hydrolysis and LC3B-RFP, and quantification of autophagosome–lysosome fusion with Ap4 hydrolysis. (**a**) HeLa cells were transfected with the autophagy sensor LC3B-RFP and cultured for 24 h (red). The cells were then treated with Ink-128, an autophagy inducer, for 4 h before electroporation with the Ap4 analog (green). Line profiles for the pixel intensities of the colocalized Ap4 and LC3B-RFP. Scale Bar: 10 µm. (**b**) Cells were transfected with LC3B-RFP (red) and cultured for 24 h. Cells were then treated with Ink-128 (green), an autophagy inducer, or chloroquine (green), an autophagy inhibitor, for 4 h before the introduction of the Ap4 analog. Quantification of intensity spots and colocalization (yellow) was done using the ComDet plugin of Fiji. Data represent the average number of fluorescent spots per cell. Error bars mark ± SD for each condition, (N ≥ 15). Statistical significance was determined by Tukey’s multiple comparisons test. Scale bar: 10 µm.

Figure 6a shows that punctuate structures of LC3B-RFP and those of the Ap4 analog were colocalized weakly, pointing at a potential cleavage of the Ap4 analog during autophagy. When the cells were treated with chloroquine, the number of LC3B-RFP puncta increased while the number of Ap4 structures decreased strongly. At the same time, the number of colocalizing LC3B-RFP and Ap4 structures remained nearly the same. This finding can be explained by the accumulation of LC3B-RFP marked autophagosomes by chloroquine treatment, which blocks the fusion between autophagic vesicles and lysosomes. This decreased Ap4 hydrolysis while the number of colocalized structures remained constant [34]. By contrast, upon induction of the autophagy by Ink-128, Ap4 hydrolysis and colocalization between the LC3B-RFP and Ap4 structures increases as compared to the untreated control. This indicates that the Ap4 analog is utilized during the later stages of autophagy (Figure 6b). Thus, our data show the potential of the newly developed Ap4 analog to follow autophagic flux in live cells.

While the identification of the enzymes cleaving the Ap4 analog is beyond the scope of this contribution, based on known lysosomal enzymes, it is possible to speculate about possible candidates. As lysosomes require a large amount of energy to maintain their low intraluminal pH of 4.2–5.3, we suspect that the Ap4 analog is cleaved by V-ATPases in processes responsible for the transport of H+ ions and the regulation of lysosomal pH [35,36]. Lysosomes are involved in a number of important cellular processes. Their membranes harbor a wide range of transporters that are involved in the active translocation of various substances like heavy metal ions and oligosaccharides. An example of this are the Cu transporters ATP7A and ATP7B, which are P-type ATPases coupling ATP hydrolysis with Cu ion transport from the cytoplasm into the lysosomes [37,38]. Lysosomal oligosaccharide transporters that play an important role in the synthesis of glycoproteins are also known to rely on ATP hydrolysis [39]. These few examples show that there are many ATP consuming lysosomal processes that could lead to the cleavage of the Ap4 analog.

## 3. Materials and Methods

### 3.1. Chemicals

Staurosporine (62996-74-1) and Ouabain Octahydrate (11018-89-6) were obtained from Sigma Aldrich (Munich, Germany). Oligomycin (1404-19-9) was purchased from Merk Millipore (Darmstadt, Germany). β-Lapachone (4707-32-8) was purchased from Cayman Chemicals (Hamburg, Germany). Bafilomycin A1 (88899-55-2) was purchased from Abcam (Berlin, Germany), and Rapamycin (53123-88-9) from AlfaAesar (Kandel, Germany). Sapanisertib (INK 128) was purchased from Cayman Chemicals (1224844-38-5) (Michigan, USA). MitoTracker™ Red CMXRos (M7512), LysoTracker Deep Red (L12492), and Premo™ Autophagy Sensor LC3B-GFP (P36235) kits were purchased from ThermoFisher Scientific (Darmstadt, Germany).

### 3.2. Fluorescence Microscopy

All fluorescent imaging experiments were performed on a TCS SP8 confocal laser scanning microscope (Leica Microsystems, Wetzlar, Germany) with a 63 X oil immersion objective lens (1.40 NA HCX PL APO CS, Leica Microsystems). The images were acquired using the LAS AF software (Leica Microsystems). For whole cell imaging, the Ap4 analog was excited by 488 nm and LysoTracker red was excited by a 552 nm wavelength. The emitted fluorescence signals were collected at 500–530 nm and 560–600nm, respectively, using the appropriate detection settings. The images were acquired with a resolution of 1024 × 1024 pixels, and were processed using ImageJ (National Institutes of Health, Bethesda, MD, USA). Fluorescence lifetime measurements were performed on a home-built time-correlated single photon counting imaging setup at RT. Then, 20 µM Ap4 analog was added into the buffer with different pH or enzyme solutions. The samples were excited by a 488nm pulsed laser (PicoQuant, Berlin, Germany) with an intensity of 10 µW. Raw data were recorded and analyzed using SymPhoTime 64 software (PicoQuant).

### 3.3. Image Processing and Statistical Analyses

All of the images were processed and all of the quantitative numbers were obtained using image analyzing software Fiji version 1.52 (National Institutes of Health). Colocalization analysis was done with Fiji’s plugin, Coloc 2. It performs the pixel intensity correlation and calculates the Pearson’s correlation coefficient. Quantification of the colocalization spots was done using Fiji’s plugin, ComDet. All data are expressed as mean ± standard deviation. Statistical analysis was done using either one-way analysis of variance (ANOVA), Tukey’s multiple comparison test, or the unpaired *t*-test for two groups, as appropriate.

### 3.4. Cell Culture and Transfection

HeLa and HEK cells were cultured in Dulbecco’s modified Eagle’s medium (DMEM, Thermo Fisher Scientific, Waltham, MA, USA) supplemented with 10% fetal calf serum (Biochrom, Cambridge, UK) and 1% penicillin/streptomycin (Thermo Fisher Scientific). The cells were maintained at 37 °C in a humidified atmosphere of 5% CO_2_. One to two days before transfection, the cells were plated at 3 × 10^4^ cells/cm3 in eight-well µ-Slides (Ibidi, Germany) for microscopy. The microscopic slides were treated with poly-L-lysine (molecular weight 70,000–150,000, Sigma Aldrich).

For the expression of the autophagy marker LC3B, HeLa cells were transiently transfected with the construct of the LC3B-red fluorescent protein using PremoTM Autophagy Sensor LC3B-RFP (Thermo Fisher Scientific, P36236), following the manufacturer’s instructions. After transfection, the cells were incubated overnight in DMEM medium for optimum LC3B expression. After overnight incubation, the Ap4 analog was incorporated into the cells for imaging.

### 3.5. Incorporation of Ap4 by Electroporation

HeLa cells were permeabilized by electroporation. First, 20,000 cells were seeded per well in eight-well μ-slides 24 h before the electroporation. Cells were washed with 1X PBS and then the Ap4 analog, diluted to 100 µM in a ZAP buffer (10 mM K_2_HPO_4_/KH_2_PO_4_ pH 7.4, 1 mM MgCl_2_, and 250 mM sucrose in water), was added. Electroporation was done using the ElectroCell S20 electroporator (βtech). Electroporation was performed at a constant voltage of 160 V with 10 pulses each for a duration of 1500 µs using parallel electrodes. The cells were washed with PBS after electroporation and the pre-warmed medium was added. For imaging, the cells were kept in DMEM without phenol red.

## 4. Conclusions

In conclusion, we have described a novel Ap4 analog that holds promise as a probe for monitoring ATP dependent enzymatic processes. The compound contains a fluorophore and a quencher, such that in its intact state, fluorescence emission is quenched. Upon cleavage, strong fluorescence emission sets result in the spatial separation of the fluorophore and quencher. Fluorescence intensity can then be employed as a read out for ATP dependent enzymatic activity. Our data show that the Ap4 analog can be internalized by cells via electroporation. It is then cleaved in live cells. Inhibition experiments clearly indicate that the compound is indeed cleaved enzymatically. Colocalization experiments based on confocal fluorescence microscopy then showed that the Ap4 analog was found in lysosomes. Further experiments indicated that autophagy might be one of the cellular processes leading to the cleavage of the Ap4 analog in live cells. This exemplifies the potential of the new compound as a probe for following ATP hydrolysis in live cells.

## Supplementary Materials

Supplementary materials on the details of the chemical synthesis and chemical analytics, as well as on stability and viability tests, are available online at https://www.mdpi.com/article/10.3390/ijms22168616/s1.
