# Supplementary material for: Live Cell Imaging of Enzymatic Turnover of an Adenosine 5′-Tetraphosphate Analog
Source: Int J Mol Sci. 2021 Aug 10;22(16):8616. doi: 10.3390/ijms22168616 (PMC8395338; doi:10.3390/ijms22168616)
Supplement: Supplementary file 1 [file ijms-22-08616-s001.zip › ijms-1302283-supplementary.pdf]

Live cell imaging of enzymatic turnover of an adenosine 5’-tetraphosphate analog

Anayat Bhat, Shuang Li, Daniel Hammler, Martin J. Winterhalder, Andreas Marx, and Andreas Zumbusch

Department Chemie, Universität Konstanz, 78457 Konstanz

Supplementary Materials

1 Experimental Section1

1.1 General information1

2 Experimental procedures4

2.1.1 N^6-(6-Aminohexyl) adenosine (2)4

2.1.2 N^6-(6-Trifluoroacetamidohexyl) adenosine (3)4

2.1.3 N^6-(6-Trifluoracetamidohexyl) adenosine-O5’-triphosphate (4)5

2.1.4 δ-(6-Azidohexyl)-N^6-(6-trifluoroacetamidohexyl) adenosine-O5’-tetraphosphate (6)6

2.1.5 Eclipse quencher 25-NHS (7)6

2.1.6 δ-(6-Azidohexyl)-N^6-(6-(Eclipse)amidohexyl) adenosine-O5’-tetraphosphate (8)7

2.1.7 δ-(6-(ATTO$_{488}$)amidohexyl)-N^6-(6-(Eclipse)amidohexyl) adenosine-O5’-tetraphosphate (9)8

3 NMR9

4 Stability ……………………………………………………………………………………………12

5 Viability tests……………………………………………………………………………………13

1 Experimental Section

1.1 General information

General Information

All reagents were used without further purification. Dry solvents were obtained from Sigma-Aldrich and used without further purification. Reactions were conducted with exclusion of air and moisture as needed. Anion-exchange chromatography was performed either on an ÄktaPurifier (GE Healthcare, USA) with a DEAE Sephadex™ A-25 (GEHealthcare Bio-SciencesAB, USA) column using a linear gradient (0.1 M – 1.0 M) of triethylammonium bicarbonate buffer (TEAB, pH 7.5) or using a Dionex DNAPac PA-100 22 x 250 mm column using 25 mM Tris-HCL pH = 8 and 5% MeCN as eluent A and 25 mM Tris-HCl pH = 8, 0.5 M $NaClO_4$ and 5% MeCN as eluent B. A linear gradient of 5% B to 40% B was applied with a flow rate of 8 mL/min. Reversed phase high pressure liquid chromatography (RP-HPLC) for the purification of compounds was performed using a Shimadzu unit having LC8a pumps and a Dynamax UV-1

detector. A VP 250/21 NUCLEODUR C18 HTec, 5 µm (Macherey-Nagel, Germany) column and a gradient of MeCN in 50 mM TEAA buffer (pH = 7.0) were used. All compounds purified by RP-HPLC were obtained as their triethylammonium salts after repeated freeze-drying. The ^{1}H NMR signals of triethylammonium are not reported. Analytical RP-HPLC was performed using a Shimadzu Prominence system. A VP 250/4 NUCLEODUR C18 Pyramid, 5 µm (Macherey-Nagel, Germany) column and a gradient of acetonitrile in 50 mM TEAA buffer (pH 7.0) were used. A linear gradient of 5% B to 50% B within 20 minutes and a flow rate of 1.0 mL/min was applied.

NMR Spectroscopy and Mass Spectrometry

^{1}H, ^{13}C, ^{19}F and ^{31}P NMR spectra were recorded in commercially available deuterated solvents on a Avance III 400 MHz spectrometer and a AVIII 600 MHz spectrometer (Bruker, Germany). All chemical shifts are given relative to the residual solvent peak and are given in ppm coupling constants are in Hz.

HR-ESI MS spectra were recorded on a Daltronics microTOF II (Bruker, Germany). The ^{1}H NMR signals of triethylammonium are not reported.

Triethylammonium bicarbonate buffer (TEAB)

1 M TEAB buffer was manufactured by suspending triethylamine (5 mol, 700mL) in water and passing carbon dioxide (from evaporated dry ice) through the mixture until the pH = 7.5. The buffer was diluted to 5 L to give 1 M TEAB. The buffer was diluted to 0.1 M as needed.

Triethylammonium acetate buffer (TEAA)

1 M TEAA buffer was obtained by mixing triethylamine (139 mL, 1 mol) with water and slowly adding acetic acid (57 mL, 1 mol). After cooling to room temperature the pH was adjusted to 7.0 and the buffer was diluted to 1 L to give 1 M TEAA and finally diluted to 50 mM as needed.

1 M $NaHCO_3$ buffer

$NaHCO_3$ (84 g, 1 mol) was dissolved in water and the pH was adjusted to pH = 8.9 or 8.7 as needed using 1 M HCl/NaOH solution. The solution was diluted to 1 L to give 1 M $NaHCO_3$ buffer.

SVPD treatment

The respective mono labelled ATP analogue (100 µM) was incubated with or without SVPD (3.3 mU/µL) in a buffer containing 100 mM NaCl, 100 mM Tris-HCl (pH = 8.7), 15 mM $MgCl_2$ and in a total volume of 30 µL for 45 min at 30 °C. For kinetic measurements (topic 3.1) the mono labelled ATP analogue (20 µM) was incubated with SVPD (0.04 mU/µL) in a buffer containing 100 mM NaCl, 100 mM Tris-HCl (pH = 8.7), 15 mM $MgCl_2$ and in a total volume of 150 µL for 15 min at 25 °C. Every 5 min 50 µL were quenched with EDTA (final conc. 0.083 mM) and analysed by analytical HPLC.

For continuously measured fluorescence lifetime the respective mono labelled ATP analogue (20 µM) was incubated with or without SVPD (0.02 mU/µl) in a buffer containing 100 mM NaCl, 100 mM Tris-HCl (pH = 8.7), 15 mM $MgCl_2$ and in a total volume of 50 µL for 60 min at 25 °C.

Absorbance/Fluorescence Spectroscopy

Absorption, emission spectra and fluorescence lifetimes were measured and determined by using standard procedures. The corresponding mono labelled ATP analogue (0.85 – 1.13 µm) was diluted in 1 x PBS buffer (pH = 7.0) to avoid the inner filter effect (absorbance = 0.1). All fluorescence experiments at ambient temperatures were performed in 10 mm polystyrene cuvettes. Absorbance spectra were recorded with a Cary 50 Bio UV-Vis spectrophotometer (Varian, USA). Fluorescence emission spectra and fluorescence lifetimes using time correlated single photon counting (TCSPS) were recorded and analysed on a FluoTime300 (PicoQuant, Germany). All fluorophores were excited at 485 nm (except $ATTO_{465}$ with a wavelength of 450 nm) using a solid-state laser-excitation source (LDH-P-C-450/485, PicoQuant, Germany). The instrument response function was recorded at the excitation wavelength using a Ludox solution. TCSPC data were analysed using Easy Tau (PicoQuant, Germany).

2 Experimental procedures

Synthesis of the Ap_4 analog

we used a hydrophilic fluorescent donor $ATTO_{488}$ with a long fluorescence lifetime of τ = 4.1 ns attached via phosphor ester to the δ-phosphate of adenosine tetra phosphate. As acceptor, a non-fluorescent quencher eclipse was synthesized and attached to the *N*6 position of the nucleobase. In this way the molecule can then be used as a turn on probe and allows us to observe the cleavage of the molecular probe by ATP hydrolyzing enzymes.

2.1.1 *N*^{6}-(6-Aminohexyl) adenosine (2)

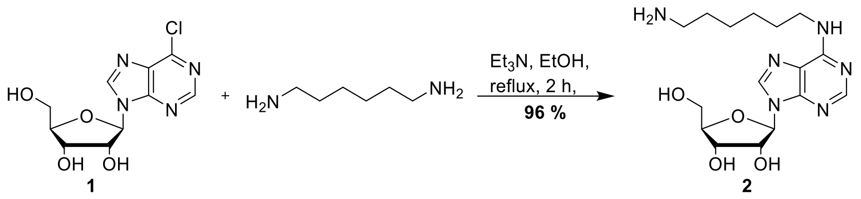

6-Chloro-9-(β-D-ribofuranosly)-purine (**1**) was prepared as previously described by Gregg *et al.* [2]. Compound **2** was prepared as previously described by Buck and Reese[3].
6-Chloro-9-(β-D-ribofuranosly)-purine (**1**) (2.00 g, 6.98 mmol, 1.0 equiv) was suspended in EtOH (60 mL), triethylamine (TEA) (0.97 mL, 0.70 g, 6.98 mmol, 1.0 equiv) and 1,6-diaminohexane (4.86 g, 41.86 mmol, 6.0 equiv) were added and refluxed for 2 hours. The reaction mixture was cooled to room temperature and precipitated for 12 hours at 4 °C. *N*^{6}-(6-Aminohexyl) adenosine (**2**) was collected by filtration to give 2.46 g (6.71 mmol, 96%).

^{1}H NMR (400 MHz, DMSO-d_6): δ = 8.33 (s, 1H, H-8), 8.19 (s, 1H, H-2), 7.86 (s, 1H, C6-NH), 5.87 (d, *J* = 6.2 Hz, 1H, H-1'), 5.42 (bs, 1H, 2'-OH), 5.41 (bs, 1H, 5'-OH), 5.17 (bs, 1H, 3'-OH), 4.61 (t, *J* = 5.6 Hz, H-2'), 4.14 (dd, *J* = 5.0, 3.0 Hz, 1H, H-3'), 3.96 (q, *J* = 3.4 Hz, 1H, H-4'), 3.67 (dd, *J* = 12.1, 3.6 Hz, 1H, H-5'a), 3.55 (dd, *J* = 12.2, 3.6 Hz, 1H, H-5'b), 3.49 - 3.40 (m, 2H, C6-NH-C**H**$_2$), 2.49 – 2.45 (m, 2H, NH_2-C**H**$_2$-linker), 1.57 (q, 2H, 1x C**H**$_2$-linker), 1.38 – 1.24 (m, 6H, 3x C**H**$_2$-linker).

^{13}C NMR (101 MHz, DMSO-d_6): δ = 154.5, 152.3, 148.0, 139.5, 119.6, 87.9, 85.8, 73.3, 70.5, 61.6, 41.5, 39.9, 33.2, 29.0, 26.2, 26.1.

2.1.2 *N*^{6}-(6-Trifluoroacetamidohexyl) adenosine (3)

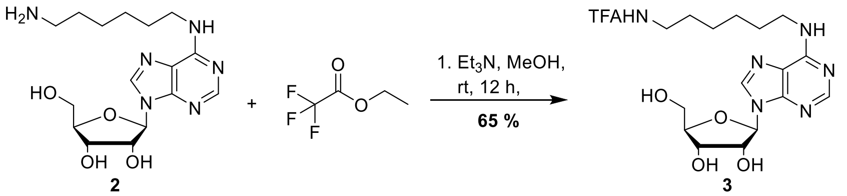

N^6-(6-Aminohexyl) adenosine (**2**) (2.40 g, 6.55 mmol, 1.0 equiv) was suspended in EtOH (70 mL). TEA (1.18mL, 0.86 g, 8.5 mmol, 1.5 equiv) and ethyl trifluoroacetate (1.17mL, 1.40 g, 9.83 mmol, 1.5 equiv) were added and the reaction mixture was stirred overnight. The product was collected by filtration, washed with MeOH (2 x 2 mL) and the filtrate was stored for another 24 hours and the precipitate again collected by filtration.
N^6-(6-Trifluoroacetamidohexyl) adenosine (**3**) was obtained as a white solid (1.97mg, 5.37 mmol, 65%).

$\varepsilon_{269\ nm}$(H_2O\DMF) = 15600 M^{-1} cm^{-1}.

^{1}H NMR (400 MHz, DMSO-d_6): δ = 9.37 (bt, 1H, NHTFA), 8.33 (s, 1H, H-8), 8.19 (bs, 1H, H-2), 7.86 (s, H, C6-NH), 5.87 (d, *J* = 6.4 Hz, 1H, H-1'), 5.45-5.36 (m, 2H, 2'-OH, 5'-OH), 5.16 (d, *J* = 4.6 Hz, 1H, 3'-OH), 4.61 (q, *J* = 5.9 Hz, 1H, H-2'), 4.14 (td, *J* = 4.8, 3.0 Hz, 1H, H-3'), 3.96 (dt, *J* = 3.4 Hz, 1H, H-4'), 3.67 (dt, *J* = 12.1, 4.1 Hz, 1H, H-5'a), 3.55 (ddd, *J* = 11.6, 7.2, 3.6 Hz, 1H, H-5'b), 3.47 (bs, 2H, C6-NH-C**H**$_2$), 3.16 (pq, *J* = 6,5 Hz, 2H, TFANH-C**H**$_2$), 1.59 (q, *J* = 7,1 Hz, 2H, C6-NH-CH$_2$-C**H**$_2$), 1.49 (p, *J* = 7,2 Hz, 2H, TFANH-CH$_2$-C**H**$_2$), 1.40 – 1.21 (m, 4H, 2x C**H**$_2$-linker).

^{13}C NMR (101 MHz, DMSO-d_6): δ = 156.1, 154.7, 152.4, 148.2, 139.6, 119.8, 115.9, 87.9, 85.9, 73.4, 70.6, 61.7, 40.1, 39.2, 28.9, 28.2, 26.0, 25.9.

^{19}F NMR (376 MHz, DMSO-d_6): δ = -74.4 (s, 3F).

2.1.3 *N*^{6}-(6-Trifluoracetamidohexyl) adenosine-O5'-triphosphate (4)

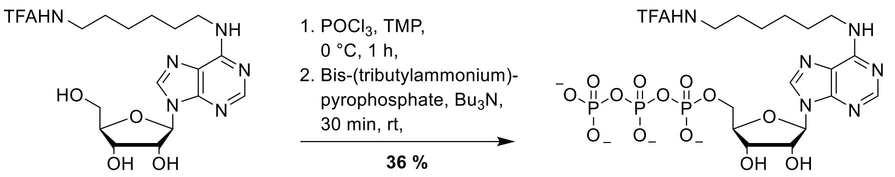

3 4

N^6-(6-Trifluoracetamidohexyl) adenosine-O5'-triphosphate (**4**) was prepared as previously described by Yoshikawa *et al.* [2].

N^6-(6-Trifluoroacetamidohexyl) adenosine (**3**) (250 mg, 540 μmol, 1 equiv) was dissolved in trimethylphosphate (TMP, 6 mL) and cooled to 0 °C. Phosphorous oxychloride (124 mg, 76.3 μL, 811 μmol, 1.5 equiv) was added dropwise to the cooled solution and kept at 0 °C for 2 hours. Tributylamine (1.03 mL, 800 mg, 4.3 mmol, 8 equiv) and bis-(tributylammonium)-pyrophosphate (1.2 g, 2.16 mmol, 4 equiv, dissolved in DMF (1.7mL)) were added and the solution was stirred at room temperature for 2 hours. The reaction was quenched by the addition of 0.1 M TEAB buffer (12 mL) and stirring for 30 minutes at room temperature. The reaction mixture was extracted three times with ethyl acetate (50 mL) and the aqueous phase was evaporated. The product was purified by anion-exchange chromatography (DEAE Sephadex™ A-25 column) and RP-HPLC. Fractions containing the product were evaporated and the product repeatedly freeze dried from water to give the triphosphate as the triethylammonium salt[4].
N^6-(6-Trifluoracetamidohexyl) adenosine-O5'-triphosphate (**4**) was obtained as a white solid (195 mmol, 36%).

^{1}H NMR (400 MHz, D$_2$O): δ = 8.46 (s, 1H, H-8), 8.15 (s, 1H, H-2), 6.07 (d, *J* = 6.0 Hz, 1H, H-1'), 4.77 – 4.70 (m, 1H, H-2'), 4.42 – 4.33 (m, 1H, H-3'), 4.24 (m, 1H, H-4'), 4.29 – 4.23 (m, 1H, H-5'a), 4.22 – 4.13 (m, 1H, H-5'b), 3.45 (bs, 2H, C6-NH-C**H**$_2$), 3.24 (t, *J* = 7.1 Hz, 2H, TFANH-C**H**$_2$), 1.61 (p, *J* = 7.4 Hz, 2H, C6-NH-CH$_2$-C**H**$_2$), 1.50 (p, *J* = 7.5 Hz, 2H, TFANH-CH$_2$-C**H**$_2$), 1.40-1.20 (m, 4H, 2x C**H**$_2$-linker).

^{19}F NMR (376 MHz, D$_2$O): δ = -75.8.

^{31}P NMR (162 MHz, D$_2$O): δ = -10.02 (d, *J* = 20.4 Hz, 1P), -11.45 (d, *J* = 20.1 Hz, 1P), -23.09 (t, *J* = 20.0 Hz, 1P).

HR MS (ESI, neg. mode): m/z: calculated for $C_{18}H_{27}F_3N_6O_{14}P_3^-$ [M-H]$^-$: 701.0745,
found: 701.0780 [M-H]$^-$, deviation: 5.0 ppm.

2.1.4 δ-(6-Azidohexyl)-N^6-(6-trifluoroacetamidohexyl) adenosine-O5'-tetraphosphate (6)

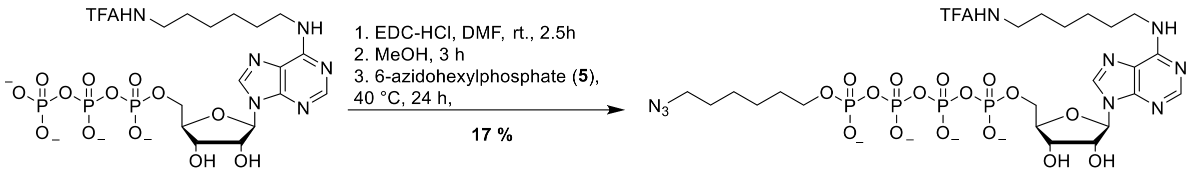

4 6

δ-(6-azidohexyl)-N^6-(6-trifluoroacetamidohexyl) adenosine-O5'-tetraphosphate (**6**) was synthesized as previously described by Hacker *et al.*[5].

N^6-(6-Trifluoroacetamidohexyl) adenosine (**4**) (400 μmol, 1 equiv) was converted into its tetrabutylammonium salt by passing it through a preequilibrated CHELEX 100 cation-exchange resin with tetrabutylammonium bromide. Compound **4** and 6-azidohexylphosphate[6] (**5**) (600 mg, 2.69 mmol, 6.7 equiv) were separately lyophilized overnight, subsequently dried in high vacuum for several hours and dissolved in dry DMF (4 mL and 5 mL). 1-Ethyl-3-(3-dimethylaminopropyl) carbodiimid hydrochloride (EDC•HCl) (230 mg, 1.2 mmol, 3 equiv) were added to compound **4** and the solution was stirred at rt for 2.5 hours. Methanol (130 μL, 3.2 mmol, 8 equiv) was added and after 3 hours Et_3N (1.1 mL, 810 mg, 8 mmol, 20 equiv) and 6-azidohexylphosphate (**5**) were added and stirred at 40 °C for 24 hours. The solvent was evaporated and the reaction was quenched with 0.1 M TEAB buffer (15 mL). The product was purified by anion-exchange chromatography (DEAE Sephadex™ A-25 column) and RP-HPLC. Fractions containing the product were evaporated and the product repeatedly freeze dried from water to give δ-(6-azidohexyl)-N^6-(6-trifluoroacetamidohexyl) adenosine-O5'-tetraphosphate (**6**) as a white solid (68.8 μmol, 17%).

^{1}H NMR (400 MHz, D_2O): δ = 8.57 (s, 1H, H-8), 8.28 (s, 1H, H-2), 6.15 (d, J = 6.3 Hz, 1H, H-1'), 4.84 (t, J = 5.7 Hz, 1H, H-2'), 4.63 (dd, J = 5.2, 3.1 Hz, 1H, H-3'), 4.42 (p, J = 2.9 Hz, 1H, H-4'), 4.37 – 4.28 (m, H-5'a), 4.28 – 4.21 (m, 1H, H-5'b), 3.94 (qd, J = 6.8, 2.3 Hz, 2H, δP-O-C**H₂**), 3.59 (s, 2H, C6-NH-C**H₂**), 3.33 (t, J = 6.9 Hz, 2H, TFANH-C**H**₂), 3.25 – 3.23 (m, 2H, N3-C**H₂**), 1.72 (p, J = 7.0 Hz, 2H), 1.60 (p, J = 7.2 Hz, 2H), 1.55 – 1.36 (m, 4H, 2x C**H₂**-linker), 1.27 – 1.16 (m, 4H, 2x C**H**₂-linker).

^{19}F NMR (376 MHz, D_2O): δ = -75.72 (s, 3F).

^{31}P NMR (162 MHz, D_2O): δ = -10.72 – -11.02 (m, 1P), -11.27 – -11.65 (m, 1P), -23.17 – -23.53 (m, 2P).

HR MS (ESI, neg. mode): m/z: calculated for $C_{24}H_{39}F_3N_9O_{17}P_4^-$ [M-H]$^-$: 906.1361,
found: 906.1367 [M-H]$^-$, deviation: 0.66 ppm.

2.1.5 Eclipse quencher 25-NHS (8)

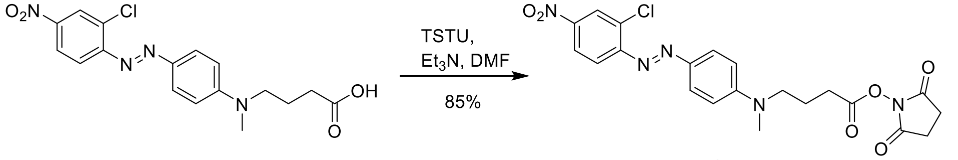

7 8

Eclipse-COOH (**7**) was synthesized according to literature[7].
Eclipse-COOH (**7**) (100 mg, 265 μmol, 1 equiv) was dissolved in DMF (3 mL), Et_3N (183 μL, 5 equiv) was added and the mixtures was cooled to 0 °C. Subsequently, *N,N,N',N'*-tetramethyl-*O*-(*N*-succinimidyl)uronium tetrafluoroborate (TSTU) (239 mg, 795 μmol) was added and the reaction mixture was allowed to warm up to room temperature and stirred for 2 hours. The product was purified by flash chromatography (DCM -> 2.5 % MeOH/DCM) and the fractions containing the product were evaporated to give Eclipse quencher 25-NHS (**8**) (107 mg, 225 μmol, 85%).

R_f = (DCM:MeOH - 93:7 v/v)) = 0.7.

^{1}H NMR (400 MHz, $CDCl_3$): δ = 8.39 (d, J = 2.4 Hz, 1H, O_2N-C-C**H**-C-Cl), 8.16 (dd, J = 8.9, 2.4 Hz, 1H, O_2N-C-C**H**-CH), 7.96 (d, J = 9.1 Hz, 2H, 2x N=N-C-C**H**), 7.78 (d, J = 8.9 Hz, 1H, O_2N-C-CH-C**H**), 6.85 – 6.71 (m, 2H, 2x Me-N-C-C**H**), 3.61 (t, J = 7.7 Hz, 3H, Me-N-C**H_2**), 3.14 (s, 3H, **Me**-N), 2.87 (s, 4H, succinimidyl), 2.71 (t, J = 6.9 Hz, 2H, CO-C**H_2**), 2.12 (p, J = 7.1 Hz, 2H, N_3-CH_2-C**H_2**).

2.1.6 δ-(6-Azidohexyl)-*N*^{6}-(6-(Eclipse)amidohexyl) adenosine-O5'-tetraphosphate (9)

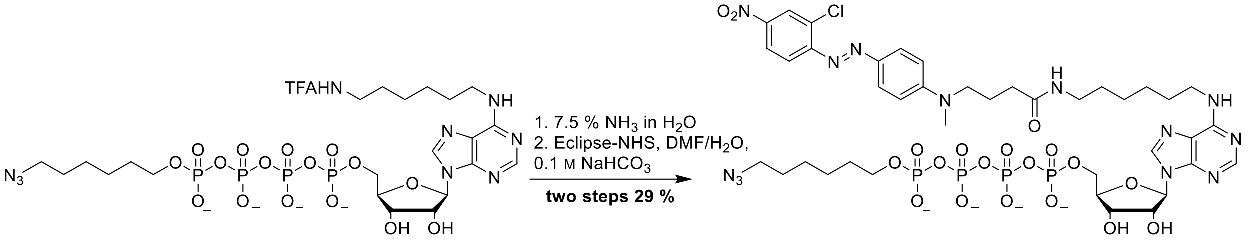

δ-(6-Azidohexyl)-*N*^{6}-(6-trifluoroacetamidohexyl) adenosine-O5'-tetraphosphate (**6**) (64 μmol, 1 equiv) was dissolved in 7.5 % NH_3 in H_2O (4.4 mL) and stirred for 5 hours at room temperature. The solvent was evaporated and the synthesis intermediate lyophilized overnight and subsequently dissolved in DMF (8 mL), H_2O (2 mL) and 1 M $NaHCO_3$ (1.1 mL). The pH was adjusted to pH = 8.7 and Eclipse-NHS (160 mmol, 75.8 mg, 2.5 equiv, dissolved in 1 mL DMF) added. The pH was kept at pH = 8.7 for 1 hour followed by evaporation of the solvents. The product was purified by RP-HPLC and the fractions containing the product were evaporated and the product repeatedly freeze dried from water to give δ-(6-azidohexyl)-*N*^{6}-(6-(Eclipse)amidohexyl) adenosine-O5'-tetraphosphate (**8**) as a purple solid (18.8 μmol, 29%).

^{1}H NMR (400 MHz, D_2O): δ = 8.49 (s, 1H, H-8), 8.18 (s, 1H, H-2), 7.77 (s, 1H, O_2N-C-C**H**-C-Cl), 7.52 (d, J = 9.0 Hz, 1H, O_2N-C-C**H**-CH), 7.34 (d, J = 8.2 Hz, 2H, 2x N=N-C-C**H**), 7.12 (d, J = 8.8 Hz, 1H, O_2N-C-CH-C**H**), 6.38 (d, J = 8.6 Hz, 2H, 2x Me-N-C-C**H**), 6.04 (d, J = 5.7 Hz, H-1'), 4.74 (d, J = 23.1 Hz, H-2'), 4.57 (t, J = 4.4 Hz, H-3'), 4.37 (bs, 1H, H-4'), 4.34 – 4.20 (m, 2H, H-5'), 3.93 (q, J = 7.0 Hz, 2H, δP-O-C**H_2**), 3.45 – 3.28 (m, 4H, C6-NH-C**H_2**, Me-N-C**H_2**), 3.18 – 3.15 (m, 2H, CONH-C**H_2**), 3.09 (t, J = 5.1 Hz, 2H, N_3-C**H_2**), 2.91 (s, 3H, **Me**-N), 2.27 (t, J = 6.3 Hz, 2H, CO-C**H_2**), 1.88 (bs, 2H, CO-CH_2-C**H_2**), 1.63 – 1.48 (m, 4H, C6-NH-CH_2-C**H_2**, N_3-CH_2-C**H_2**), 1.42 (q, J = 7.4 Hz, 4H, linker), 1.22 (s, 8H, linker).

^{31}P NMR (162 MHz, D_2O): δ = -10.87 (dt, *J* = 19.8, 7.2 Hz, 1P), -11.09 – -11.87 (m 1P), -23.26 (td, *J* = 19.4, 7.0 Hz, 1P).

HR MS (ESI, neg. mode): m/z: calculated $C_{39}H_{54}ClN_{13}O_{19}P_4^{2-}$ $[M-2H]^{2-}$: 583.6143,
found: 583.6152 $[M-2H]^{2-}$, deviation: 1.54 ppm.

2.1.7 δ-(6-(ATTO$_{488}$)amidohexyl)-*N*^{6}-(6-(Eclipse)amidohexyl) adenosine-O5’-tetraphosphate (10)

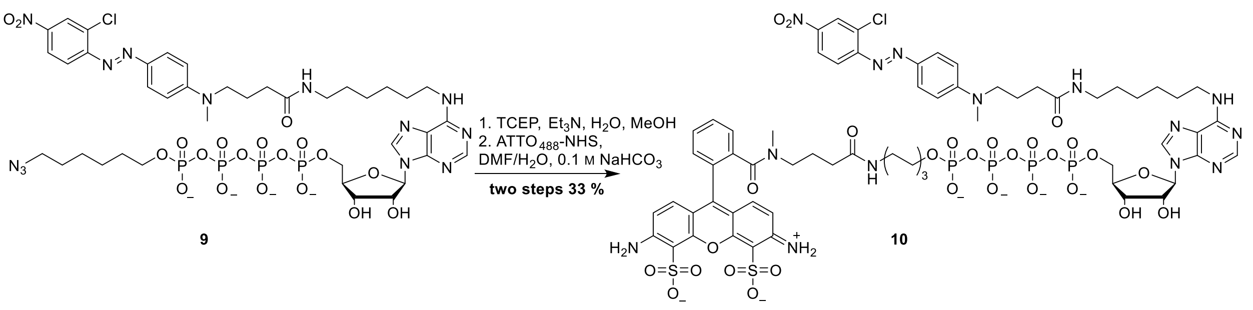

δ-(6-Azidohexyl)-*N*^{6}-(6-(Eclipse)amidohexyl) adenosine-O5’-tetraphosphate (**9**) (18.8 μmol, 1 equiv) was dissolved in a mixture of Et_3N (4 mL), H_2O (7 mL) and MeOH (8 mL). TCEP tris(2-carboxyethyl) phosphine (27 mg, 94 μmol, 5 equiv) was added and the reaction mixture was stirred overnight at room temperature. Another portion of TCEP (10.8 mg, 37.6 μmol, 2 equiv) was added and stirred at room temperature for additional 3 hours. The product was purified by RP-HPLC and the fractions containing the product were evaporated and the intermediate product repeatedly freeze dried from water. Subsequent, the intermediate product with a free amine was dissolved in H_2O (1mL) and a 1 M $NaHCO_3$ (111 μL) solution was added. The pH was adjusted to pH = 8.7 and ATTO$_{488}$-NHS (12.9 mg, 18.8 μmol, 1 equiv) was added in 3 portion while the pH was always controlled and adjusted to pH = 8.7. After 2 hours the mixture was purified by IEX (Dionex DNAPac PA-100 column) followed by RP-HPLC. The solvent was evaporated and the product repeatedly freeze dried from water to give δ-(6-(ATTO$_{488}$)amidohexyl)-*N*^{6}-(6-(Eclipse)amidohexyl) adenosine-O5’-tetraphosphate (**10**) (5.95 μmol, 33%).

^{1}H NMR (600 MHz, D_2O, 2 rotamers): δ = 8.52 (s, ≈ 0.5H, 1. rotamer, H-8), 8.46 (s, ≈ 0.5H, 1. rotamer, H-8), 8.09 (s, ≈ 0.5H, 1. rotamer, H-2), 7.99 (s, ≈ 0.5H, 2. rotamer, H-2), 7.87 – 7.63 (m, 3H, H-5, H-6, O_2N-C-C**H**-C-Cl), 7.62 – 7.15 (m, 6H, H-4, H-7, O_2N-C-C**H**-CH, O_2N-C-CH-C**H**, 2x N=N-C-C**H**), 6.88 – 6.48 (m, 4H, H-1’,H-8’, H-2’, H-7’), 6.48 – 6.32 (m, 2H, 2x N-C-C**H**), 5.99 (pseudo dd, *J* = 11.2, 5.8 Hz, 1H, H-1’), 4.69 (bs, 1H, H-2’), 4.61 – 4.46 (m, 1H, H-3’), 4.38 – 4.30 (m, 1H, H-4’), 4.30 – 4.13 (m, 2H, H-5’), 3.99 (t, J = 7.8, 3.6 Hz, ≈ 1.2H, 1. rotamer, γ-PO_4-C**H**$_2$), 3.86 (d, J = 6.8 Hz, ≈ 0.8H, 2. rotamer, γ-PO_4-C**H**$_2$), 3.43 – 3.29 (m, 2H, C6-NH-C**H$_2$**), 3.14 – 2.90 and 2.87 – 2.78 (m, 7.2H (rotamers), 2x Me-N-C**H**$_2$, 2x CO-NH-C**H**$_2$), 2.88 (s, 3H, Me-N (Eclipse)), 2.68 – 2.61 (≈ 0.8H, 2. rotamer, Me-N (ATTO$_{488}$)) 2.57 (s, ≈ 1.5 H, 1. rotamer, Me-N (ATTO$_{488}$)), 2.45 (s, ≈ 1.5 H, 2. rotamer, Me-N (ATTO$_{488}$)), 2.22 (bs, 2H, CO-C**H$_2$**), 1.98 – 1.91 and 1.91 – 1.76 (m, 3H, C6-NH-CH$_2$-C**H**$_2$, CO-C**H$_2$**), 1.68 (bs, ≈ 1H, 2. rotamer, CO-C**H$_2$**), 1.64 – 1.53 (m, ≈ 1H, 1. rotamer, γ-PO_4-CH$_2$-C**H**$_2$, ≈ 1H, 2. rotamer, Me-N-CH$_2$-C**H**$_2$), 1.44 – 1.26 (m, 4H, 2. rotamer, γ-PO_4-CH$_2$-C**H**$_2$, linker), 1.26 – 1.20 (m, 6H, linker), 1.33 – 1.27 (m, 10H, linker).

^{31}P NMR (162 MHz, D_2O): δ = -10.83 (1P), -11.41 (1P), -23.21 (2P).

HR MS (ESI, neg. mode): m/z: calculated $C_{64}H_{76}ClN_{14}O_{28}P_4S_2^{2-}$ $[M-3H]^{3-}$: 570.4350,
found: 570.4369 $[M-3H]^{3-}$, deviation: 3.3 ppm.

3 NMR

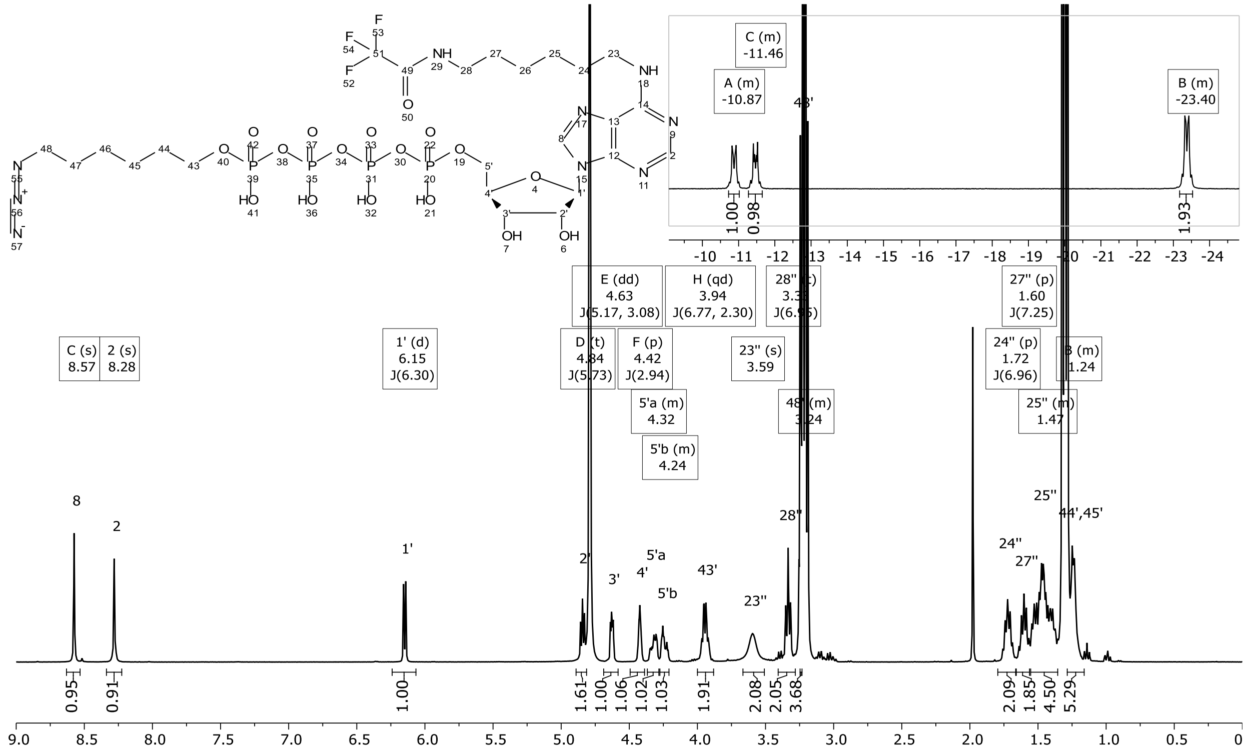

^1H NMR (400 MHz, D_2O): δ-(6-Azidohexyl)-*N*6-(6-trifluoroacetamidohexyl) adenosine-O5’-tetraphosphate (6)
Inlet: ^{31}P NMR (162 MHz, D_2O). Eclipse quencher 25-NHS (**6**)

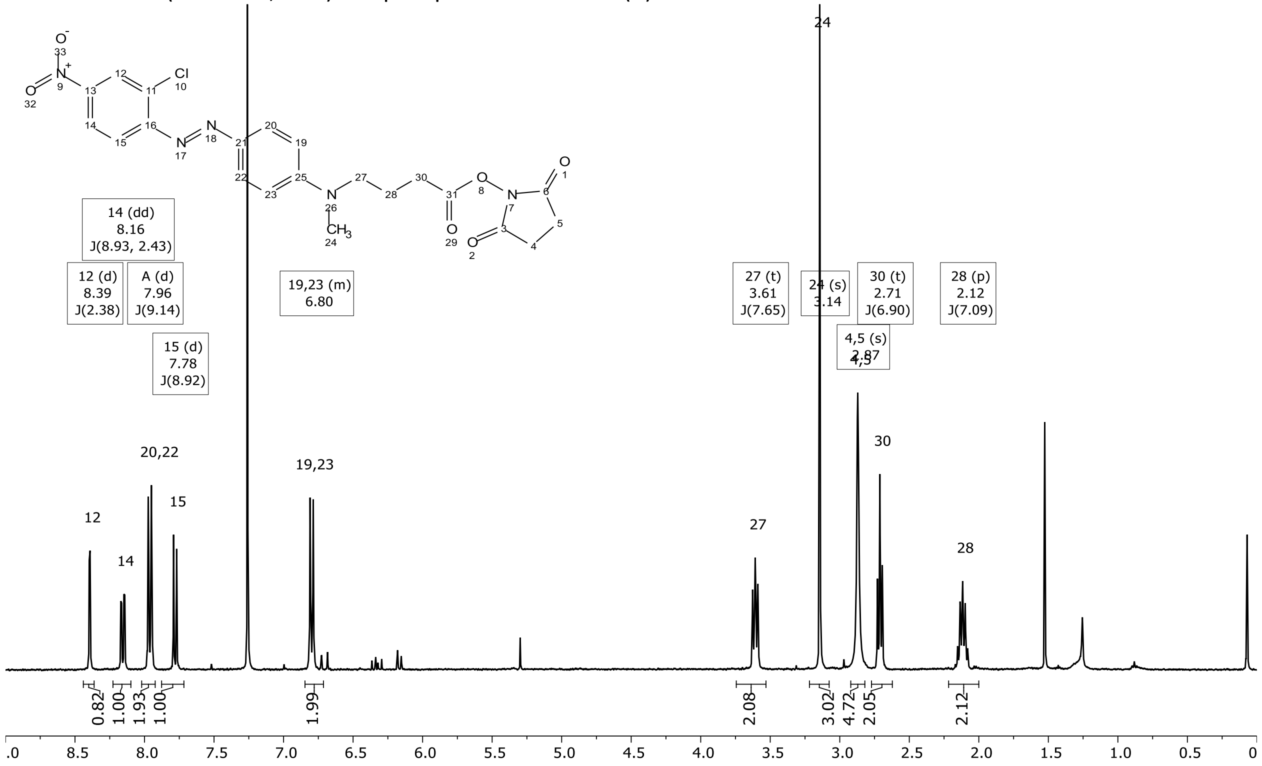

^1H NMR (400 MHz, $CDCl_3$): Eclipse quencher 25-NHS (**8**)

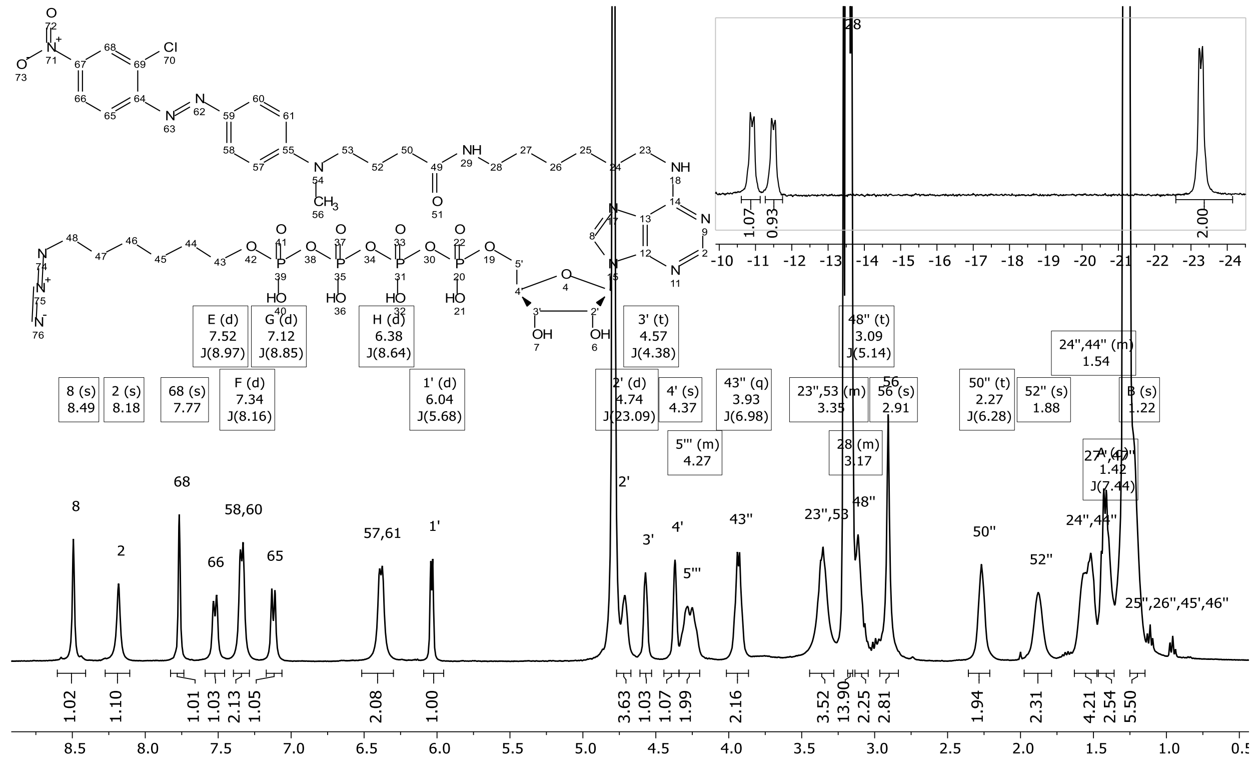

^{1}H NMR (400 MHz, D_2O): δ-(6-Azidohexyl)-*N*^{6}-(6-(Eclipse)amidohexyl) adenosine-O5′-tetraphosphate (**9**)
Inlet: ^{31}P NMR (162 MHz, D_2O).

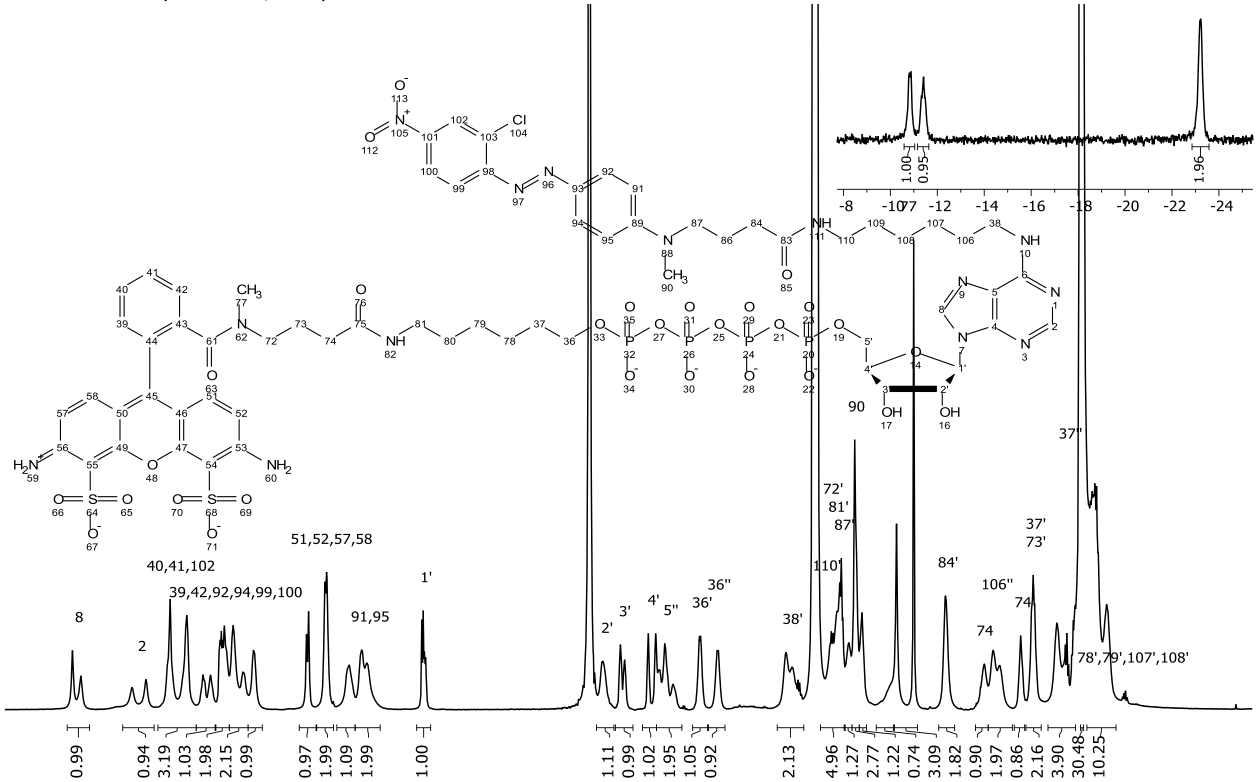

^{1}H NMR (600 MHz, D_2O): δ-(6-(ATTO$_{488}$)amidohexyl)-*N*^{6}-(6-(Eclipse)amidohexyl) adenosine-O5′-tetraphosphate (**10**)
Inlet: ^{31}P NMR (162 MHz, D_2O).

4 Stability

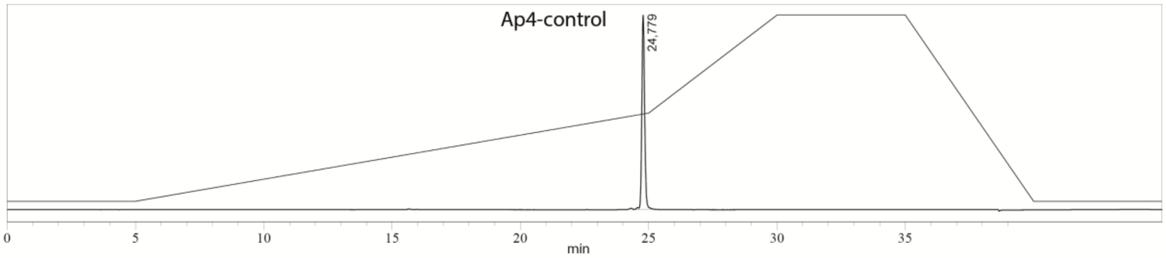
Ap4-control
24,779
0 5 10 15 20 25 30 35
min

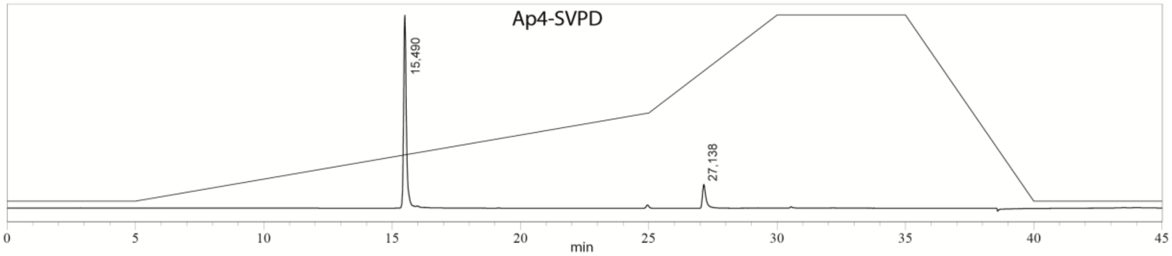
Ap4-SVPD
15,490
27,138
0 5 10 15 20 25 30 35 40 45
min

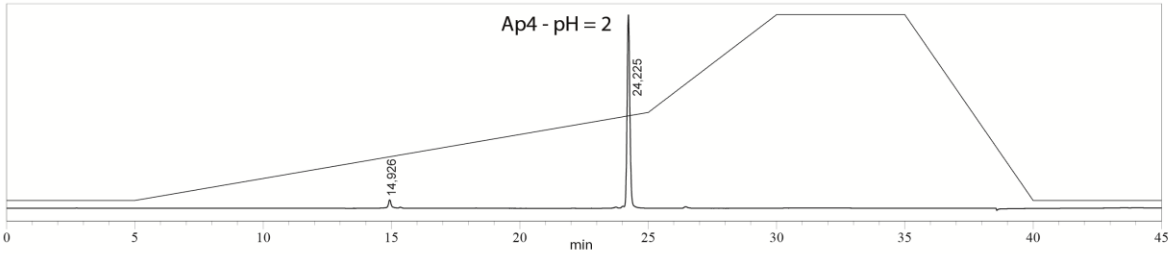
Ap4 - pH = 2
24,225
14,926
0 5 10 15 20 25 30 35 40 45
min

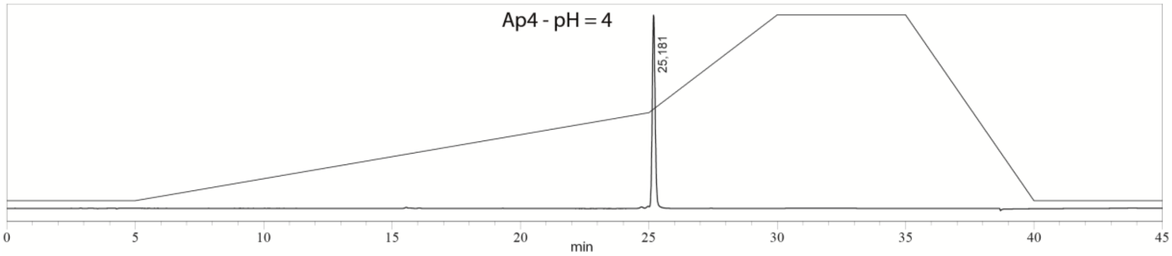
Ap4 - pH = 4
25,181
0 5 10 15 20 25 30 35 40 45
min

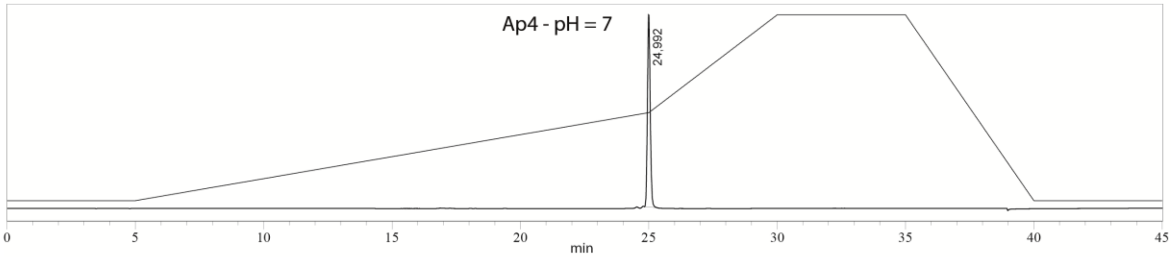
Ap4 - pH = 7
24,992
0 5 10 15 20 25 30 35 40 45
min

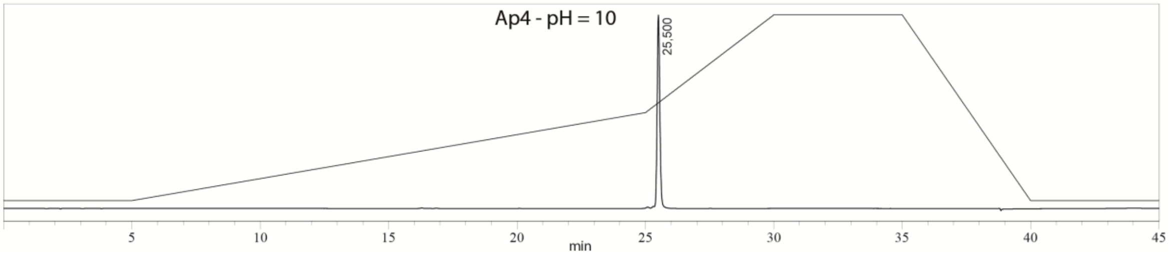
Ap4 - pH = 10
25,500
5 10 15 20 25 30 35 40 45
min

With our target compound in hand, we tested the stability for different pH values. To ensure a stable pH value during the experiments, we used for pH = 2 and 10, a glycine, for pH = 4, an acetate and for pH = 7, a phosphate buffer with a final concertation of 50 mm and incubated compound 2 for 2.5 hours at 37 °C. Analytical HPLC revealed that only for pH = 2 a small amount of compound 2 (4%) was hydrolyzed while incubation time (analytical HPLC runs are shown in section 4). For all other pH values no degradation over time was observed showing that the synthesized ATP analogue is stable over a wide range of pH and thus suitable for in cellulo studies.

5 Cell viability

Cell viability assay was performed using Alamar-Blue(AB) cell viability reagent (ThermoFisher Scientific). It is a resazurin-based solution that measures the reducing power of the living cells to give a quantative estimation of the cell health and viability. The redox reaction is accompanied by the change in colour of the culture media that is quantified by colourimeter or fluorimeter.

HeLa cells were cultured in 8-well ibidi slides in complete DMEM at 37° C in presence of 5% CO_2 overnight. The cells were electroporated in presence of the different concentrations of Ap_4 analog as described. After two hours of incubation, the cells were treated with the Alamar Blue reagent as per the manufacturer's guidelines. AB reagent added to only culture media without cells was used as blank. Cells without electroporation and Ap_4 treatment was used as a control. After the required incubation as per the protocol, the culture media with the AB reagent was transferred to a 96-well plate and read using a standard plate reader with the emission filter of 590/35 nm. The values of AB reduction of treated cells were normalized to that of control cells to calculate the percentage of viability. It was observed that there is not a considerable reduction in the viability even at the higher concentrations of Ap_4 as compared to the concentrations we are using in the time scale at which we are doing the experiments. All the experiments were performed in triplicates in three independent assays.

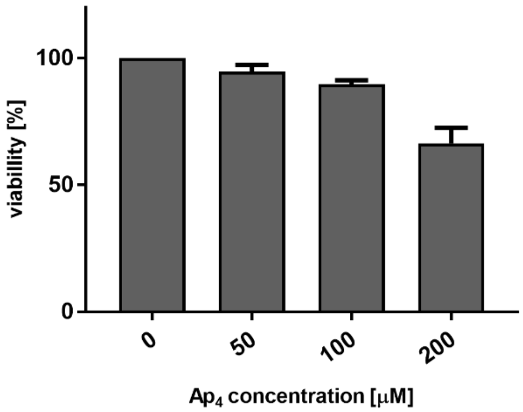

6 References

[1] F. Doll, A. Buntz, A.-K. Späte, V. F. Schart, A. Timper, W. Schrimpf, C. R. Hauck, A. Zumbusch, V. Wittmann, *Angew. Chem. Int. Ed* **2016**, *55*, 2262-2266.

[2] I. M. Buck, C. B. Reese, *Journal of the Chemical Society, Perkin Transactions 1* **1990**, 2937-2942.

[3] A. Gregg, S. E. Bottle, S. M. Devine, H. Figler, J. Linden, P. White, C. W. Pouton, V. Urmaliya, P. J. Scammells, *Bioorganic \& Medicinal Chemistry Letters* **2007**, *17*, 5437-5441.

[4] M. Yoshikawa, T. Kato, T. Takenishi, *Bull. Chem. Soc. Jpn.* **1969**, *42*, 3505-3508.

[5] S. M. Hacker, M. Mex, A. Marx, *The Journal of Organic Chemistry* **2012**, *77*, 10450-10454.

[6] M. Mex, *AG Prof. Andreas Marx* **2011**, Bachelor Thesis.

[7] a) J. M. Heemstra, J. S. Moore, *Tetrahedron* **2004**, *60*, 7287-7292; b) G. W. Dombrowski, J. P. Dinnocenzo, P. A. Zielinski, S. Farid, Z. M. Wosinska, I. R. Gould, *The Journal of Organic Chemistry* **2005**, *70*, 3791-3800.
